# A techno-economic investigation of conventional and innovative desiccant solutions based on moisture sorption analysis

**DOI:** 10.1016/j.heliyon.2023.e18825

**Published:** 2023-08-01

**Authors:** Alessandro Giampieri, Yngrid Machado, Janie Ling-Chin, Anthony Paul Roskilly, Zhiwei Ma

**Affiliations:** Department of Engineering, Durham University, Durham, DH1 3LE, United Kingdom

**Keywords:** liquid desiccant, Surfactant and nanoparticles, Ionic liquids, Moisture absorption and desoprtion, Techno-economic analysis

## Abstract

Liquid desiccant technology is an energy-efficient substitute for technologies that are conventionally applied for temperature and humidity control; however, innovative desiccant solutions have not been extensively explored in terms of their performance and feasibility. This work aimed to investigate desiccant solutions with moisture sorption analysis technically and economically. Various conditions of temperature and humidity were tested in a climatic chamber and the moisture absorption and desorption capacity, thermo-chemical energy storage capacity, and cost of conventional and innovative desiccant solutions were assessed by experiment. Calcium chloride showed the highest moisture desorption capacity (0.3113 g_H2O_/g_sol_ in the climatic chamber at 50 °C and 25% RH) and the lowest cost, despite its low moisture absorption capacity. Ionic liquids show high moisture absorption capacity (as high as 0.429 g_H2O_/g_sol_ in the climatic chamber at 25 °C and 90% RH) and could be used as additives (in which a maximum increase of 84.1% was observed for moisture absorption capacity due to the addition of ionic liquids), and thus, they are promising substitutes for conventional desiccant solutions. As solutions for better performance under various conditions were identified, the study will advance liquid desiccant technology.

## Nomenclature

*[BMIM][BF*_*4*_*]*: 1-butyl-3-methylimidazolium tetrafluoroborate
*[DMIM][OAc]*: 1,3-dimethylimidazolium acetate
*[EMIM][OAc]*: 1-ethyl-3-methyl imidazolium acetate
*[EMIM][MeSO*_*3*_*]*: 1-ethyl-3-methylimidazolium methanesulfonate
*[EMIM][BF*_*4*_*]*: 1-ethyl-3-methylimidazolium tetrafluoroborate
*[Ch][DMPO*_*4*_*]*: 2-hydroxy-N,N,N-trimethylethan-1-aminium dimethyl phosphate
*Al*_*2*_*O*_*3*_: aluminium oxide
*CaCl*_*2*_: calcium chloride
*CNT*: carbon nanotube
*CuO*: copper oxide
*IL*: ionic liquid
*Fe*: iron
*Fe*_*2*_*O*_*3*_: iron oxide
*LiBr*: lithium bromide
*LiCl*: lithium chloride
*MWNT*: multi-walled carbon nanotube
*NP*: nanoparticle
*PVP*: polyvinylpyrrolidone
*HCO*_*2*_*K*: potassium formate
*SiO*_*2*_: silicon dioxide
*[P*_*4441*_*][DMPO*_*4*_*]*: tributyl(methyl)phosphonium dimethyl phosphate

**Variables**
*ω*: moisture content (g_H2O_/kg_dry.air_)
*x*: mass fraction (g_salt_/g_sol_)
*MAC*: moisture absorption capacity (g_H2O_/g_sol_)
*MDC*: moisture desorption capacity (g_H2O_/g_sol_)
*C*: cost (£)
*M*: mass (g)
*T*: temperature (°C)
*RH*: relative humidity (%)

**Subscript**
*eq*: equilibrium of the solution
*dry air*: air
*dil*: diluted solution
*conc*: concentrated solution
*sol*: solution
*sp*: specific
*THC*: temperature and humidity chamber
*H*_*2*_*O*: water

## 1 Introduction

Liquid desiccant technology exploits the affinity to water molecules of hygroscopic solutions, which can be used for a variety of applications, such as moisture control and removal, industrial drying at low temperatures, *etc.* [1]. Lithium chloride (LiCl), calcium chloride (CaCl_2_) and other hygroscopic aqueous solutions of halide salts are common working fluids for these systems, despite of their drawbacks such as being corrosive and prone to crystallisation [2]. Moreover, aqueous LiCl solution is expensive whereas aqueous CaCl_2_ solution cannot give satisfactory dehumidification performance for deep dehumidification processes. Aqueous potassium formate (HCO_2_K) solutions have been studied as a less corrosive alternative to aqueous solutions of halide salts because of their low corrosivity, low potential for crystallisation and good dehumidification performance [3]. Mixtures of desiccant salts have also been studied to maintain optimal performance at a reduced cost [[4], [5], [6]]. To date, solutions with surfactants added, nanofluids and ionic liquids have been explored as innovative solutions for liquid desiccant application for higher performance, reduced corrosiveness, evitable crystallisation, and lower cost.

Nanofluids are engineered fluids that are obtained by suspending particles with sizes ranging 1–100 nm in water, oil, ethylene glycol, and other base fluids*.* Nanoparticles (NPs) used in nanofluids are carbon materials, metals, graphite, graphene, diamond, metal oxides, graphene oxide, *etc*. [7]. When NPs are added to desiccant solutions, this results in an increase in interactions and collisions between the NPs and the desiccant solution, which increases the solution's turbulence, fluctuations and heat and mass transfer [8]. In addition, nanofluids employing metal NPs (*e.g.*, gold and silver) and metal oxides NPs (*e.g.*, copper oxide (CuO), zinc oxide (ZnO), aluminium oxide (Al_2_O_3_), titanium oxide (TiO_2_), magnesium oxide (MgO), *etc.*) could enhance the sanitising property of conventional liquid desiccant solutions [9] due to their ability to inhibit the activity of fungi, viruses, and bacteria [10]. Ali et al. [11,12] found no significant effect on the performance when ultrafine particles of copper (Cu) were added to an aqueous CaCl_2_ solution in a falling film dehumidification system. Kang et al. [13] observed increased mass transfer where the best performance was obtained for carbon nanotube (CNT) NPs (up to 2.48 times higher than the base fluid for a concentration of the NPs of 0.1% wt.) when iron (Fe) and CNT NPs (0.01–0.1% wt.) were added in a falling film dehumidification system using aqueous LiBr solution as the desiccant solution. Kim et al. [14] mixed silicon dioxide (SiO_2_) NPs (0.01–0.1% vol.) with an aqueous LiBr solution in a falling film dehumidifier by using ultrasonic disruption and magnetic stirring techniques. The surfactant 2E1H with a concentration of 150 ppm was added to the solution. Heat and mass transfer increased by 46.8% and 18% for 0.005% vol. SiO_2_. Langroudi et al. [15] reported a 12.23% and 13.22% increase in heat and mass transfer when gamma-phase aluminium oxide (γ-Al_2_O_3_) NPs (0.02% wt.) were added to an aqueous LiBr solution. The nanofluid was prepared by using ultrasonic disruption and magnetic stirring techniques. Wen et al. [16,17] observed an increase in the dehumidification and regeneration rate of 25.9% and 24.7%, respectively, when multi-walled carbon nanotube (MNWT) (0.1% wt.) was tested in a falling film internally cooled dehumidifier and internally heated regenerator where surfactant polyvinylpyrrolidone (PVP–K30) (0.4% wt.) was added for stabilisation. Talal [18] simulated the increase in performance due to the addition of the NPs (Al_2_O_3_, Cu and SiO_2_) in counter and parallel flow falling film dehumidifiers, identifying an increase in the dehumidification performance due to the addition NPs that depends on the flow configuration of the dehumidification unit. Shoaib et al. [8] investigated the increase in performance due to the addition of CuO NPs (0.35% vol.) in a counter flow packed bed dehumidifier, showing increased mass transfer (average rate of 4.5 g/m^2^·sec). The nanofluid was prepared using an ultrasonic bath technique for 3 h. Zheng et al. [19] investigated the increase in performance due to the addition of SiO_2_ NPs (1% and 2% wt.) in a gas-liquid hollow fibre membrane dehumidifier, identifying an increase in the absorption performance that is proportional to the concentration of NPs in the nanofluid (up to 2.39 times of the base fluid for 2% wt. NP). In addition, the nucleation sites for crystal growth could be increased by adding NPs [20], which could be beneficial in absorption-based thermal batteries for thermo-chemical energy storage. Lin et al. [20] reported enhanced heat and cold storage density by 24.8% and 156%, respectively, when SiO_2_ NPs mixed with a LiCl crystallised slurry were tested.

The stability of nanofluids is a major problem for their application in liquid desiccant systems. The key requirements for the use of nanofluids are (i) to produce an even, stable and durable suspension, (ii) with a negligible agglomeration of particles and (iii) no chemical changes in the fluid [21]. The stability of the nanofluid could be enhanced by adding surfactants to it (in addition to the possibility of adding the surfactant to the stand-alone conventional desiccant solution) [22]. For application in “open” systems, the surfactant is required to be non-volatile, odourless and non-toxic. As reviewed by Wen and Lu [22], most of the surfactants added to the working fluids in absorption refrigeration or heat pump systems present small to high toxicity and odours and, as such, their use is not feasible in liquid desiccant systems. On the other hand, PVP-K30 is a water-soluble polymer, hygroscopic, characterised by good adhesive properties and stable pH, which represents a suitable candidate for the mixture with desiccant solutions [22]. When PVP-K30 was added to an aqueous LiCl solution for both the dehumidification and the regeneration process, Wen et al. [23,24] found that the increase in the concentration of the surfactant in the LiCl solution would reduce the contact angle on a stainless steel plate before levelling off. In a falling film internally cooled or heated system, their results showed that by adding PVP-K30 (which enlarged the wetting area whilst reducing the falling film thickness), dehumidification and regeneration rates would be increased by up to 22.7% and 26.3%, respectively. Hu et al. [25] added PVP-K30 to an aqueous LiBr solution for volatile organic compounds (VOCs) removal and found that the use of PVP-K30 would enhance both the solubility of hydrophobic VOCs in the desiccant solution as well as mass transfer at the solution interface.

Ionic liquids (ILs) were investigated as replacements for conventional desiccant solutions due to their solubility at ambient temperature (*i.e.* no problems of crystallisation), low or negligible corrosiveness and very low vapour pressure (*i.e.* very high dehumidification performance) [2]. In comparing the performances of 1-ethyl-3-methylimidazolium tetrafluoroborate ([EMIM][BF4]) (83.2% wt.) and aqueous LiBr solution (45% wt.) for a counter-flow dehumidifier, Luo et al. [26] found that the [EMIM][BF_4_] aqueous solution showed a lower dehumidification rate by about 13%. When the performances of 1-butyl-3-methylimidazolium tetrafluoroborate ([BMIM][BF_4_]) (85.5% wt.), 1,3-dimethylimidazolium acetate ([DMIM][OAc]) (81.3% wt.), aqueous LiCl (40.9% wt.), and LiBr solution (45% wt.) in a counter-flow dehumidifier were compared, Luo et al. [27] reported a similar dehumidification rate for the ILs and the conventional desiccant fluids where the best performance was shown by [DMIM][OAc] among the ILs. Qu et al. [28] calculated the thermo-physical properties useful for air-conditioning applications (such as the equilibrium vapour pressure, the specific heat capacity, the density and the dynamic viscosity) of 1-ethyl-3-methyl imidazolium acetate ([EMIM][OAc]). By analysing the dehumidification capacity of ILs (16 potential options in total), Watanabe et al. [29] found that tributyl (methyl)phosphonium dimethyl phosphate ([P_4441_][DMPO_4_]) (77% wt.) has the best performance because of its high dehumidification capacity, low corrosiveness and stability. Turnaoglu et al. [30] investigated a fibre bundle membrane dehumidifier using Sorbionic04, a mixture of 1-ethyl-3-methylimidazolium methanesulfonate ([EMIM][MeSO_3_]) with benzotriazole as corrosion inhibitor, as the desiccant solution, and the experimental results showed a good performance in terms of dehumidification effectiveness and compactness of the system. By investigating the dehumidification capacity of 7 potential ILs, Maekawa et al. [31] found that 2-hydroxy-N,N,N-trimethylethan-1-aminium dimethyl phosphate ([Ch][DMPO4]) (80% wt.) would show the best performance due to its high dehumidification capacity, low corrosiveness, low cost and stability. By investigating the operation of a membrane-based heat and mass exchanger, Wang et al. [32] found reduced membrane contamination and increased durability of the dehumidifier when [EMIM][OAc] was used as the working fluid.

It is clear that several studies have investigated the use of innovative fluids in liquid desiccant systems — most of these studies focused on the performance of stand-alone solutions with limited studies comparing their performance with that of conventional desiccant solutions. In addition, most of the studies focused on the dehumidification process rather than the regeneration one (which could be beneficial for the investigation of the low-grade heat recovery capacity of desiccant solutions), showing the knowledge gap in the research on innovative fluids for liquid desiccant application. The literature review also showed that none of the studies has evaluated the cost and thermo-chemical energy storage capacity of using innovative fluids in liquid desiccant systems. As such, this study aims to investigate and compare the use of conventional and innovative desiccant solutions for the dehumidification and regeneration process by using a moisture sorption analysis, which is a technique that has been previously applied to adsorption processes in solid materials [33,34] instead of desiccant solutions. The objectives are (i) understanding the moisture absorption and desorption behaviour of conventional and innovative fluids with experiments; and (ii) assessing and comparing the feasibility of using innovative fluids from a techno-economic point of view. This study could be a base for developing liquid desiccant systems which requires the identification of the solutions for better performance under various conditions.

In this article, Section 2 presents the method applied to the study. Section 3 describes the procedure that was used to prepare the conventional and innovative desiccant solutions and the experimental setup for the moisture sorption analysis. Section 4 shows and discusses the results of the moisture absorption and desorption analysis, including the economic analysis of the use of conventional and innovative fluids and highlighting additional factors that must be considered when desiccant solutions are used for dehumidification and thermo-chemical energy storage in liquid desiccant systems.

## 2 Methodology

**Fig. 1:**
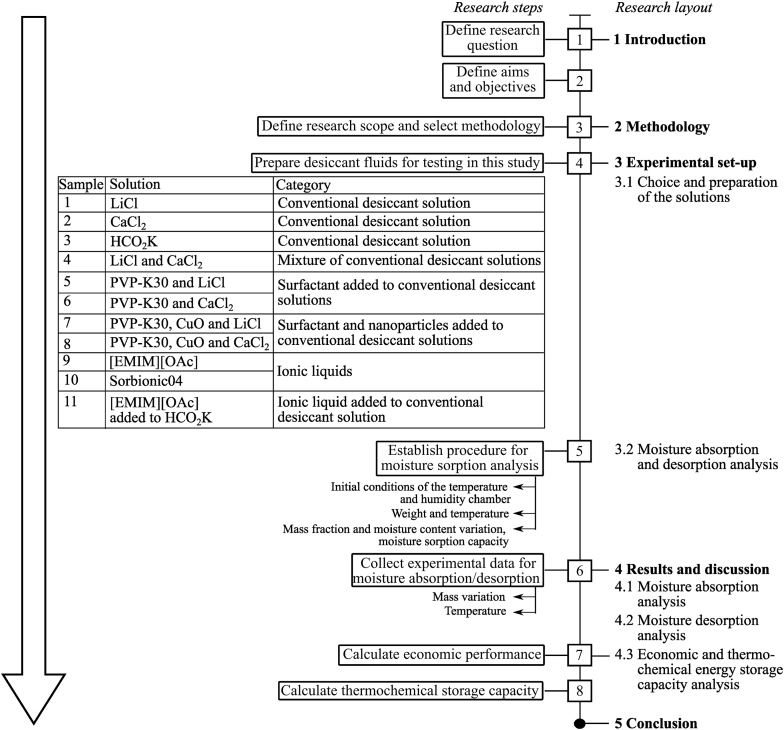
Methodology applied for the techno-economic investigation based on moisture sorption analysis of conventional and innovative solutions used for liquid desiccant application.

The methodology developed for the study is illustrated in Fig. 1, aiming to develop an approach that would enable the comparison of conventional and innovative desiccant solutions from a techno-economic point of view and help identify innovative desiccant solutions that could substitute for conventional desiccant solutions in the near future.

## 3 Experimental setup

### 3.1 Choice and preparation of the solutions

Table 1 summarises the characteristics of the desiccant salts (*i.e.*, LiCl, CaCl_2_, and HCO_2_K), surfactants (*i.e.*, PVP-K30), NPs (*i.e.*, CuO) and ILs (*i.e.*, [EMIM][OAc] and Sorbionic04) investigated in this study and why they were chosen. The mixtures of LiCl with CaCl_2_ were investigated, while the mixtures of LiCl and CaCl_2_ with HCO_2_K were deselected as they would produce a chalky solution, which is not suitable for application in liquid desiccant air-conditioning systems.

**Table 1:** Characteristics of the desiccant salts, surfactants, NPs and ILs used in the study.

Component	Characteristics	Supplier	Justification of choice	Ref.
LiCl	Hygroscopic anhydrous salt (purity 99.3%) in the form of white crystalline powder	Leverton Lithium	Conventional desiccant solution	[35]
CaCl_2_	White, flaked, hygroscopic dihydrate salt with 77% wt. CaCl_2_	Tetra	Conventional desiccant solution	[36]
HCO_2_K	Soluble in water and very hygroscopic, purity 99% wt. (water content less than 2%)	Fisher Scientific	Conventional desiccant solution	[37]
PVP-K30	White to yellowish-white hygroscopic powder; 0.4% wt. PVP-K30 offers an optimal concentration in a desiccant solution [23,24]	Thermo Fisher Scientific	Non-volatile, odourless and non-toxic	[38]
CuO	Black coloured NPs with size of 30–40 nm and a specific surface area of 29 m^2^/g	Merck Life Science UK Limited	Antimicrobial; beneficial for application in highly occupied areas	[39,40]
[EMIM][OAc]	Colourless to slightly yellow fluid, purity higher than 98% wt.	Proionic	High capacity for dehumidification [28], low corrosiveness [41], relatively low dynamic viscosity [42] and solubility at ambient temperature [43]	[44]
Sorbionic04	Slightly turbid yellow fluid	Proionic	High thermal stability and solubility at ambient temperature [43]	[44,45]

**Fig. 2:**
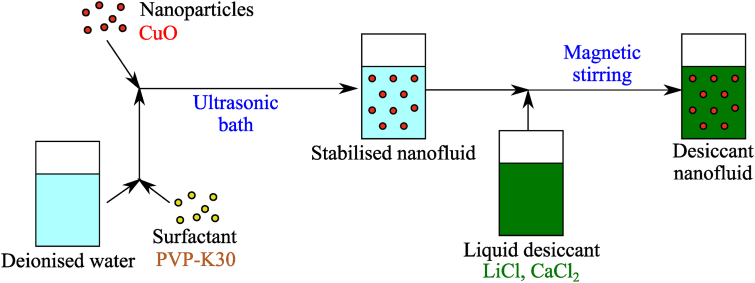
Preparation of desiccant-based nanofluids with a two-step method, based on [14].

Solutions required for experiments were prepared as described here. LiCl, CaCl_2_, HCO_2_K, and PVP-K30 were solid crystals; as such, they were mixed with deionised water to achieve the percent by weight (% wt.) of each solution set for the experiments. Metal oxide-based nanofluids can be prepared in 2 ways, either the one-step or two-step method [7]: the one-step method involves a simultaneous synthesis and dispersion of metal oxide NPs in a base fluid; on the other hand, the two-step method involves the synthesis of metal oxide NPs first, followed by subsequent dispersion in the base fluid. The modified two-step method, as shown in Fig. 2, was selected for this study as it allowed the use of commercially available NPs *i.e.*, CuO, to produce a satisfactory nanofluid for experiments. Ultrasonication was applied to intensify the dispersion of the CuO NPs into the base fluid and avoid their agglomeration [21]. A surfactant *i.e.*, PVP-K30 (which alters the surface properties of the suspended particles and reduces the tendency of particles to stick together) was added to optimise the dispersion of the NPs in the solution [21]. Whilst the concentration of PVP-K30 in deionised water was selected based on [23,24], 1% wt. PVP-K30 was also tested. For ILs, [EMIM][OAc] and Sorbionic04 manufactured by Proionic appeared in a liquid form. Therefore, pure and water-mixed [EMIM][OAc] and Sorbionic04 were tested in this study.

**Fig. 3:**
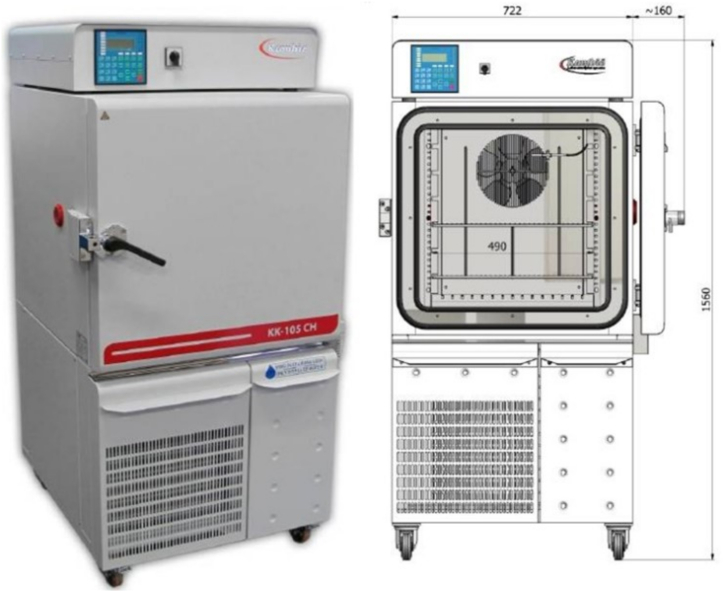
The temperature and humidity chamber used for the moisture absorption and desorption analysis [46].

### 3.2 Moisture absorption and desorption analysis

The following procedure was applied for the moisture absorption and desorption analysis.

1) A temperature and humidity chamber (Manufacturer: Kambic, Model KK-105 CH with a temperature range of 5–180 °C and an RH range of 10–98%), as shown in Fig. 3, was used in this study to run experiments for moisture absorption and desorption analysis. The temperature and RH inside the chamber were fixed to meet the conditions set for the moisture absorption and desorption processes, as summarised in Table 2.

**Table 2:** Experimental conditions set for the moisture absorption and desorption analysis.

	Condition	*T*_THC_ (°C)	*RH*_THC_ (%)	*ω*_THC_ (g_H2O_/kg_dry air_)	Number of experiments
Moisture absorption process	1	25	70	12.3	20
	2	18	95	13.9	49
	3	25	90	18	43
Moisture desorption process	1	40	35	16.3	10
	2	50	25	19.5	25
	3	45	35	21.3	24
	4	70	15	30.1	18
	5	90	15	72.1	12
2) Samples of solutions were prepared where their temperatures were recorded. The weight of the samples was measured using a digital scale (Model Kern DS 150K1 with precision ±0.02 g).
3) Variations in temperature due to the moisture absorption and desorption processes were determined using a K-type thermocouple linked to a PicoLog TC-08 thermocouple data logger [56].
4) Variations in mass fraction, *x*_*sol*_, were then calculated. In addition, for conventional desiccant solutions, the variations in equilibrium moisture content, *ω*_*eq*_, of the solutions were calculated. The correlations for the equilibrium moisture content of LiCl and CaCl_2_ were obtained from Ref. [53], while that of HCO_2_K was found in Ref. [54].
5) The moisture absorption capacity, *MAC* (g_H2O_/g_sol_), and the moisture desorption capacity, *MDC* (g_H2O_/g_sol_), were calculated using Eqs. (1), (2) [57]:

(1) $$MAC=\frac{M_{f}-M_{i}}{M_{i}}$$

(2) $$MDC=\frac{M_{i}-M_{f}}{M_{i}}$$

where *M*_*f*_ and *M*_*i*_ represent the final and initial mass (g) of a sample after the moisture sorption process, respectively.

6) The economic performance of each solution was determined based on the costs of MAC and MDC, *C*_*MAC*_ (g_H2O_/£) and *C*_*MRC*_ (g_H2O_/£), using Eqs. (3), (4):

(3) $$C_{MAC}=\frac{M_{f}-M_{i}}{C_{M_{i}}}$$

(4) $$C_{MDC}=\frac{M_{i}-M_{f}}{C_{M_{i}}}$$

where *C*_*Mi*_ is the cost (£) of the desiccant salts, surfactants, NPs and/or ILs used in preparing the solution, as shown in Table 3 [[47], [48], [49], [50], [51], [52]].

7) The thermo-chemical energy storage potential of the desiccant solutions was compared based on the volumetric energy storage density, *ESD* (kWh/m^3^), as defined by Ref. [58] and reported in Eq. (5):

(5) $$ESD=\rho_{dil}x_{dil}\left(\frac{1-x_{conc}}{x_{conc}}-\frac{1-x_{dil}}{x_{dil}}\right)\left(h_{fg}\right)$$

where *ρ*_*dil*_ is the density of the diluted desiccant solution (kg/m^3^), *x*_*dil*_ and *x*_*conc*_ are the mass fractions of the diluted and concentrated desiccant solutions (kg_salt_/kg_sol_), respectively, and *h*_*fg*_ is the latent heat of evaporation of water (kJ/kg). For the calculation, the density of aqueous solutions is obtained from literature: LiCl and CaCl_2_ [53]; HCO_2_K [54]; [EMIM][OAc] [28]; Sorbionic04 [45] (considering [EMIM][MeSO_3_] as fluid). The additive rule presented in Ref. [55] was applied to determine the density of mixtures.

**Table 3:** Cost of desiccant salts, surfactants, NPs and/or ILs used in the study.

Material	C (£/kg)
LiCl	52.24
CaCl_2_	0.146
HCO_2_K	0.97
PVP-K30	8.82
CuO	123.96
[EMIM][OAc]	20.27
Sorbionic04	18.46

## 4 Results and discussion

### 4.1 Moisture absorption analysis

#### 4.1.1 Conventional desiccant solutions

**Fig. 4:**
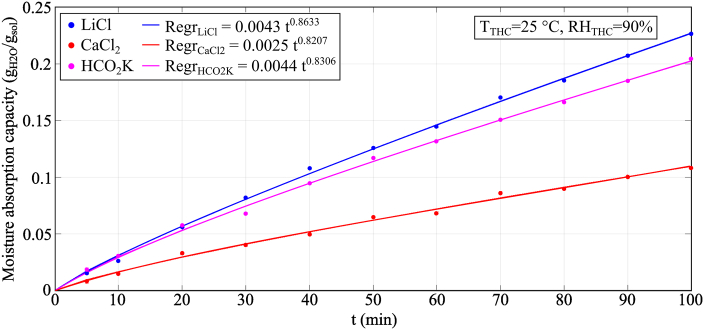
Experimental results of the conventional desiccant fluids for moisture absorption analysis.

Fig. 4 illustrates the moisture absorption capacity of three aqueous solution samples of LiCl (30.2% wt.), CaCl_2_ (31% wt.) and HCO_2_K (61.9% wt.) when the temperature and humidity chamber was set as 25 °C and 90% RH. The highest moisture absorption performance was shown by LiCl, followed by HCO_2_K and CaCl_2_. The moisture absorption process and the equilibrium moisture content of a desiccant solution, *ω*_eq_, are in an inverse relationship: the lower the equilibrium moisture content, the higher the moisture absorption performance (*ω*_LiCl_ = 7.59 g_H2O_/kg_dry air_ < *ω*_HCO2K_ = 7.74 g_H2O_/kg_dry air_ < *ω*_CaCl2_ = 10.83 g_H2O_/kg_dry air_).

**Fig. 5:**
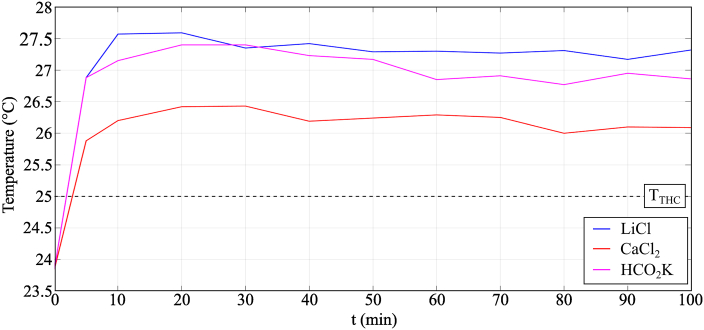
Temperature variation of the samples during moisture absorption process in Condition 3 (*T*_THC_ = 25 °C and *RH*_THC_ = 90%).

**Fig. 6:**
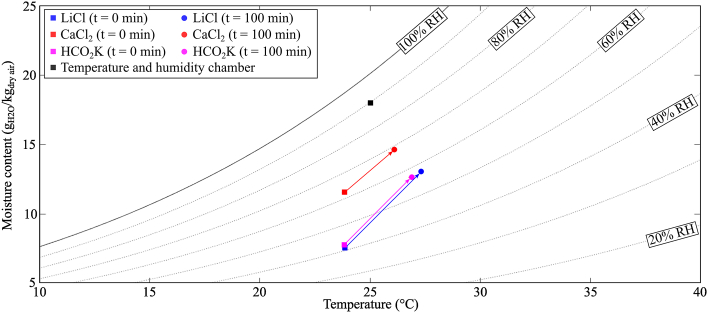
Representation on a psychrometric chart of the moisture absorption process in Condition 3 (*T*_THC_ = 25 °C and *RH*_THC_ = 90%).

Fig. 5 shows the temperature variation of the samples during the experiment for the same condition of the temperature and humidity chamber, *i.e.*, 25 °C and 90% RH. When the desiccant solutions were placed in the temperature and humidity chamber, their initial temperatures were the same as the room temperature. At the start of the experiment, these solutions absorbed moisture from the surrounding air resulting in an increase in the temperature of each solution due to the latent heat of condensation. During the experiment, moisture absorbed by each solution from the air decreased over time because the solution was diluted by water absorption and consequently became less capable of absorbing moisture from the air, which resulted in a slight decrease in the temperature of the solution. As implied by the psychrometric chart in Fig. 6, the moisture absorption process would be driven by the difference between the equilibrium moisture content of the desiccant solution, *ω*_eq_, and the moisture content of the air in the temperature and humidity chamber, *ω*_THC_. During moisture absorption, *ω*_eq_ increased as the desiccant solution absorbed moisture from the surrounding air and became diluted; the rate of moisture absorption by the desiccant solution reduced until, after sufficient time, absorption stopped as the solution reached a state of equilibrium. In this study, the largest difference between *ω*_eq_ and *ω*_THC_ was observed for the aqueous LiCl solution, which offered the best performing moisture absorption process compared to aqueous CaCl_2_ and HCO_2_K solutions.

**Fig. 7:**
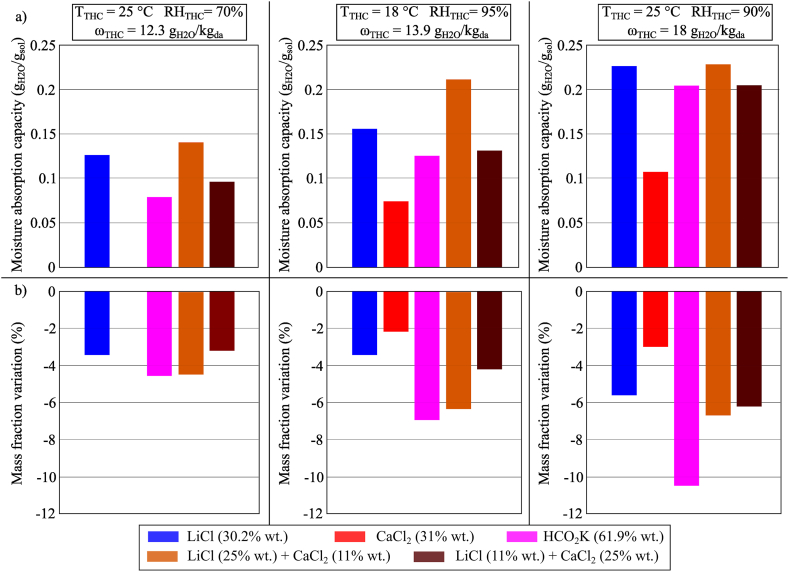
(a) MAC and (b) mass fraction variation of conventional desiccant solutions and mixtures during the moisture absorption process after 100 min in Conditions 1 (*T*_*THC*_ = 25 °C, *RH*_*THC*_ = 70%), 2 (*T*_*THC*_ = 18 °C, *RH*_*THC*_ = 95%), and 3 (*T*_*THC*_ = 25 °C, *RH*_*THC*_ = 90%).

The moisture absorption performance of LiCl, CaCl_2_, HCO_2_K and mixtures of LiCl and CaCl_2_ (for two different ratios) were investigated in three conditions of the temperature and relative humidity in the climatic chamber, and the results are illustrated in Fig. 7(a). The best moisture absorption performance was shown by the mixture of LiCl/CaCl_2_ (25% wt./11% wt.), followed by LiCl (30.2% wt.), mixture of LiCl/CaCl_2_ (11% wt./25% wt.), and HCO_2_K (61.9% wt.), in descending order. CaCl_2_ (31% wt.) showed no moisture absorption in Condition 1 which has the lowest *ω*_THC_ *i.e*., 12.3 g_H2O_/kg_dry air_ compared to other conditions. As such, CaCl_2_ should be deselected for deep dehumidification processes.

Fig. 7(b) shows the changes in the mass fraction of the investigated samples at the start and the end of the experiment. The magnitude is negative as the solution was diluted by the absorption process. In this study, HCO_2_K was the only solution working in the “water poor” mass fraction zone (*i.e.* the concentration of the water in the solution is lower than 50%). Desiccant samples operating with a water mass fraction lower than 50% would produce a larger mass fraction change between the beginning and the end of the moisture absorption process for the same amount of moisture absorbed. This affected the dehumidification capacity and thermo-chemical energy storage of the desiccant solution, as further described in Section 4.3.

Also implied in Fig. 7(a and b), an increase in *ω*_THC_ would enhance the moisture absorption process performance. When CaCl_2_ was used for moisture absorption, the sample was unable to absorb moisture from the air in Condition 1 (with the lowest *ω*_THC_); it absorbed moisture in Condition 2 (MAC_CaCl2_ = 0.0743 g_H2O_/g_sol_) and its performance was further improved in Condition 3 (MAC_CaCl2_ = 0.1079 g_H2O_/g_sol_). This trend was also shown by LiCl and HCO_2_K.

#### 4.1.2 Addition of surfactant

The addition of PVP-K30 would reduce the surface tension of the desiccant solution, which could be beneficial to the use in liquid desiccant systems by (i) creating a surface tension gradient at the interface between air and desiccant solution, which increases the mass transfer (this phenomenon is also known as the Marangoni effect [59,60]), and (ii) reducing the contact angle of the desiccant solution, resulting in an increase of the wetting ratio and a decrease of the film thickness. Desiccant systems with thin film could improve the moisture absorption process performance (*i.e.* dehumidification).

**Fig. 8:**
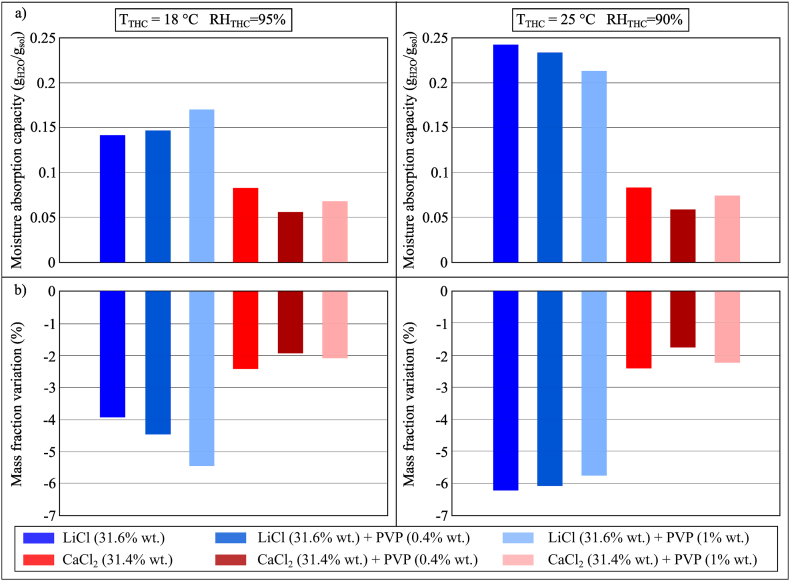
(a) MAC and (b) mass fraction variation of conventional desiccant solutions and PVP-K30 during the moisture absorption process after 100 min in Conditions 2 (*T*_THC_ = 18 °C, *RH*_THC_ = 95%) and 3 (*T*_THC_ = 25 °C, *RH*_THC_ = 90%).

The moisture absorption performance of LiCl (31.6% wt.) and CaCl_2_ (31.4% wt.) with and without PVP-K30 (0.4% wt. and 1% wt.) were compared, as shown in Fig. 8(a and b). However, these results indicated that the addition of PVP-K30 did not have a significant effect on the moisture absorption process; the type of base desiccant fluid would determine the moisture absorption capacity of the investigated sample. The moisture absorption capacity of LiCl samples was found higher than that of CaCl_2_ samples. This could be due to the fact that the moisture absorption analysis is mainly ruled by the equilibrium vapour pressure and moisture content of the desiccant solution (as previously mentioned in Section 4.1.1) or to the inability of the testing method to capture the benefits of adding the surfactant to the desiccant.

#### 4.1.3 Addition of nanoparticles

**Fig. 9:**
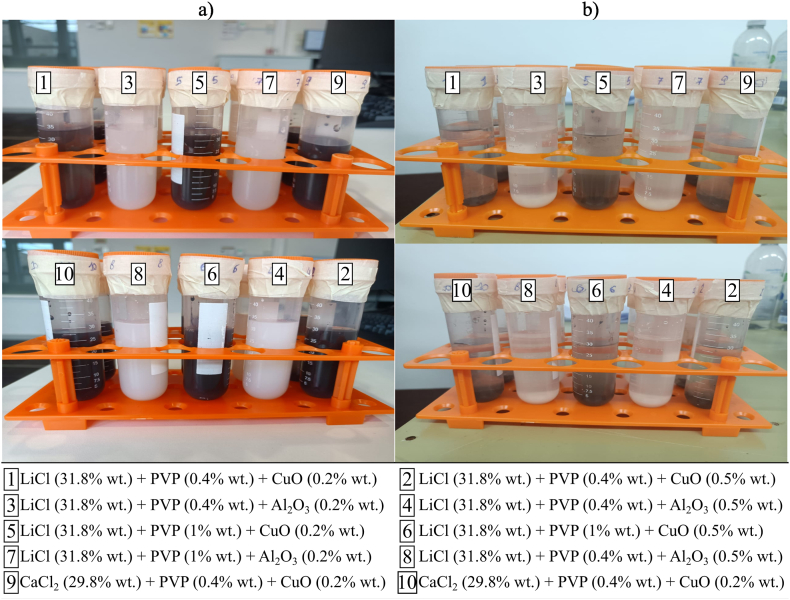
Nanofluids prepared for the moisture absorption analysis. Photos were taken (a) immediately after preparation and (b) 6 h after preparation.

**Fig. 10:**
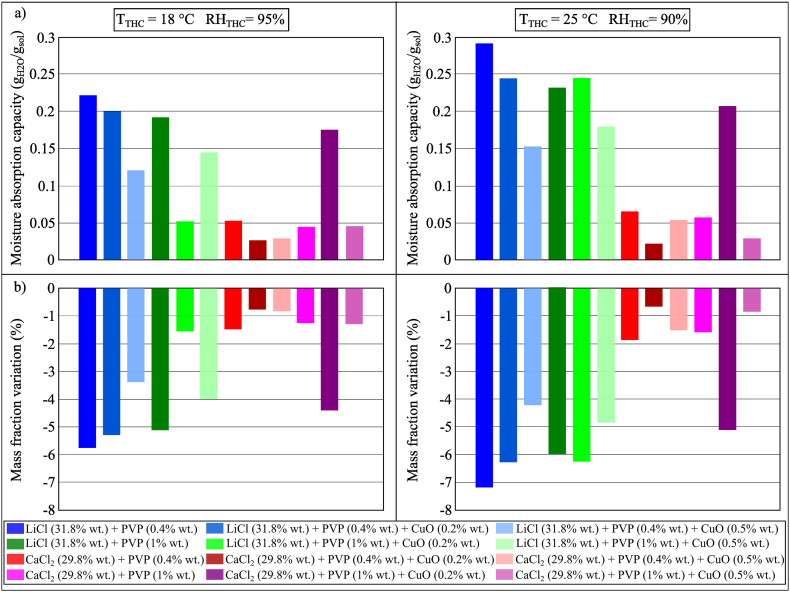
(a) MAC and (b) mass fraction variation of conventional desiccant solutions, PVP-K30 and CuO during the moisture absorption process after 100 min in Conditions 2 (*T*_THC_ = 18 °C, *RH*_THC_ = 95%), and 3 (*T*_THC_ = 25 °C, *RH*_THC_ = 90%).

The desiccant-based nanofluids prepared for the analysis, *i.e.*, PVP-K30 (0.4% or 1% wt.) with and without CuO NPs (0.2% or 0.5% wt.) added to LiCl or CaCl_2_, are shown in Fig. 9(a) after preparation and in Fig. 9(b) after 6 h. The produced samples indicated that the CuO-based nanofluids showed suspension instability. As illustrated in Fig. 10(a and b), the results showed that when CuO NPs were added to the mixture of LiCl or CaCl_2_ and PVP-K30, the performance of the moisture absorption process for all samples, except for the mixture of CaCl_2_ (*x* = 31.6% wt.), PVP-K30 (*x* = 1% wt.) and CuO (*x* = 0.2% wt.), would reduce. The moisture absorption performance of the mixture of CaCl_2_ (*x* = 31.6% wt.), PVP-K30 (*x* = 1% wt.) and CuO (*x* = 0.2% wt.) is comparable with that of the LiCl samples. This shows that the increase in performance due to the addition of NPs is possible but the stability of the nanofluid may have a primary role in determining the moisture absorption capacity of a nanofluid. Similarly, a significant decrease in the moisture absorption performance of the samples using LiCl as the base desiccant salt could be attributable to the instability of the nanofluid.

#### 4.1.4 Ionic liquids

**Fig. 11:**
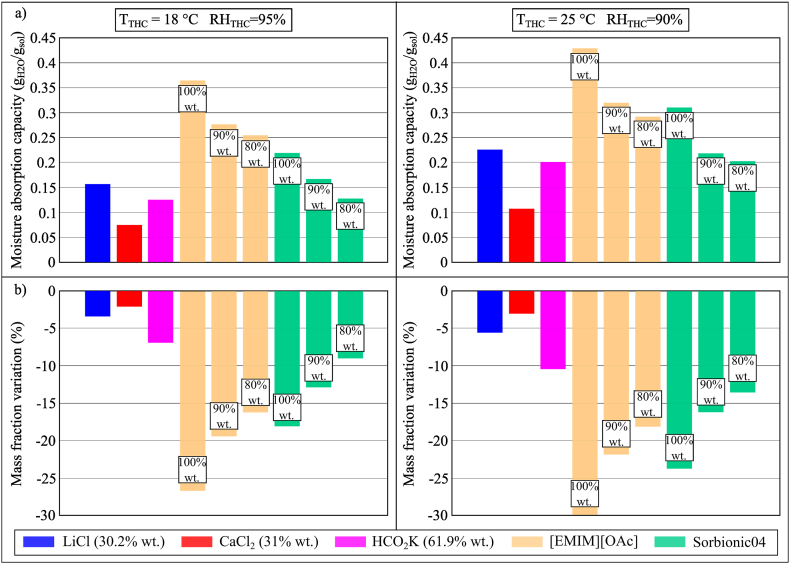
(a) MAC and (b) mass fraction variation of conventional desiccant solutions, [EMIM][OAc], and Sorbionic04 during the moisture absorption process after 100 min in Conditions 2 (*T*_THC_ = 18 °C, *RH*_THC_ = 95%) and 3 (*T*_THC_ = 25 °C, *RH*_THC_ = 90%).

The moisture absorption performance of two ILs, [EMIM][OAc] (80% wt., 90% wt., and 100% wt.) and Sorbionic04 (80% wt., 90% wt., and 100% wt.), were compared to LiCl (30.2% wt.), CaCl_2_ (31% wt.) and HCO_2_K (61.9% wt.), as shown in Fig. 11(a and b). Whilst both [EMIM][OAc] and Sorbionic04 showed a better performance in the moisture absorption process compared to LiCl, CaCl_2_ and HCO_2_K, [EMIM][OAc] (100% wt.) showed the highest moisture sorption capacity in the study, reaching 0.429 g_H2O_/g_sol_ in the climatic chamber at 25 °C and 90% RH. Sorbionic04 showed a lower dehumidification performance than [EMIM][OAc]. In both Conditions 2 and 3 of the chamber, the moisture absorption capacity of Sorbionic04 (80% wt.) *i.e.*, 0.1265 g_H2O_/g_sol_ and 0.204 g_H2O_/g_sol_, respectively, was similar to that of HCO_2_K (61.9% wt.) *i.e.*, 0.126 g_H2O_/g_sol_ and 0.2038 g_H2O_/g_sol_, respectively, but lower than that of LiCl (30.2% wt.) *i.e.*, 0.156 g_H2O_/g_sol_ and 0.226 g_H2O_/g_sol_.

**Fig. 12:**
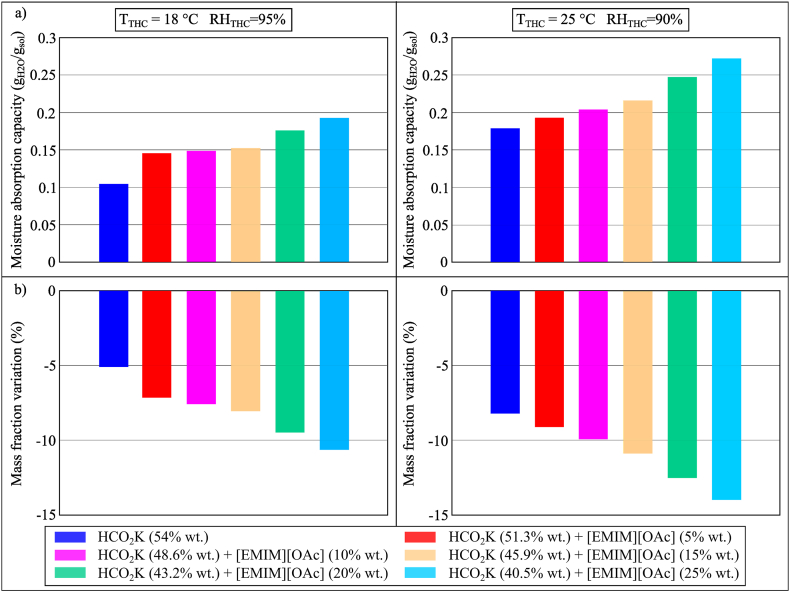
(a) MAC and (b) mass fraction variation of HCO_2_K and the mixtures of HCO_2_K and [EMIM][OAc] during the moisture absorption process after 100 min in Conditions 2 (*T*_THC_ = 18 °C, *RH*_THC_ = 95%) and 3 (*T*_THC_ = 25 °C, *RH*_THC_ = 90%).

In assessing the use of ILs as an additive, mixtures of LiCl, CaCl_2_ and HCO_2_K with [EMIM][OAc] were assessed. The mixtures of LiCl and CaCl_2_ with [EMIM][OAc] were deselected in this study as they produced a chalky solution. HCO_2_K and [EMIM][OAc] mixed well and the results in Conditions 2 (*T* = 18 °C, *RH* = 95%) and 3 (*T* = 25 °C, *RH* = 90%) were compared with that of aqueous HCO_2_K solution, as shown in Fig. 12(a and b). When the concentration of [EMIM][OAc] increased from 0% wt. (aqueous HCO_2_K solution) to 25% wt., its MAC would also increase significantly (0.1044 g_H2O_/g_sol_ vs. 0.1922 g_H2O_/g_sol_ and 0.1793 g_H2O_/g_sol_ vs. 0.2706 g_H2O_/g_sol_ in Conditions 2 and 3, respectively). This indicated the potential of adding [EMIM][OAc] to an aqueous HCO_2_K solution for deep dehumidification. Whilst HCO_2_K/[EMIM][OAc] would not present problems in terms of crystallisation, its corrosiveness should be further investigated.

### 4.2 Moisture desorption analysis

#### 4.2.1 Conventional desiccant solutions

**Fig. 13:**
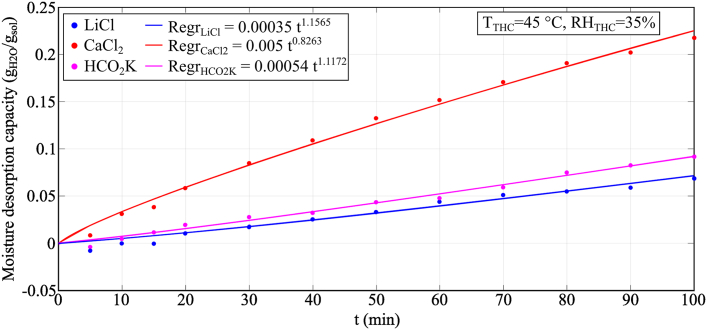
Experimental results of LiCl, CaCl_2_ and HCO_2_K during the moisture desorption analysis.

Fig. 13 shows the moisture desorption capacity of LiCl (30.2% wt.), CaCl_2_ (31% wt.) and HCO_2_K (61.9% wt.) for Condition 3 of the temperature and humidity chamber (*T* = 45 °C, *RH* = 35%). The highest moisture performance is shown by CaCl_2_, followed by HCO_2_K and LiCl. The moisture desorption process was affected directly by the equilibrium moisture content of the desiccant solution, *ω*_eq_: the higher the equilibrium moisture content, the higher the moisture absorption performance (*ω*_CaCl2_ = 10.83 g_H2O_/kg_dry air_ > *ω*_HCO2K_ = 7.74 g_H2O_/kg_dry air_ > *ω*_LiCl_ = 7.59 g_H2O_/kg_dry air_). In this experiment, CaCl_2_ showed a very high moisture desorption capacity (0.228 g_H2O_/g_sol_) when the sample was 43.1 °C. This indicated the higher capacity of CaCl_2_ to be regenerated by lower temperature heat sources, which could enhance the use of desiccant solutions to recover low-grade heat sources at about or slightly lower than 40 °C, such as hot water from compressed air units [61]. On the other hand, LiCl and HCO_2_K would require a temperature higher than 45 °C to provide a desorption process with good performance. Fig. 13 presented negative values of MDC for the LiCl and HCO_2_K samples in the first 15 min of the experiments (*i.e.*, moisture absorption rather than moisture desorption took place). As previously shown in Fig. 4, the desiccant solution samples would absorb moisture from the air straight away when experiments were started at room temperature. Unlike the moisture absorption process, the desiccant samples would need to reach a temperature higher than 40 °C at the start of the experiments to desorb moisture to the air in the climatic chamber.

**Fig. 14:**
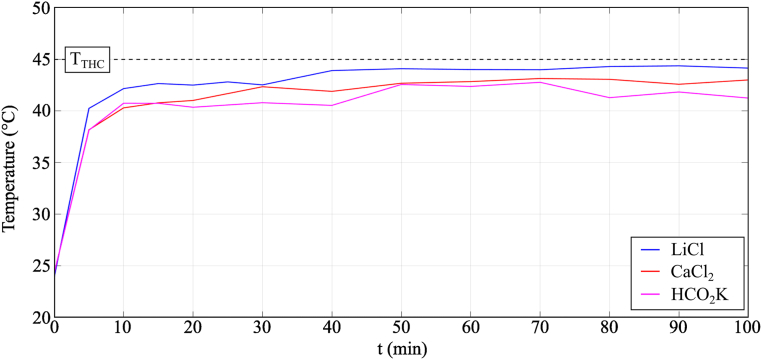
Temperature variation of the samples during moisture desorption process in Condition 3 (*T*_THC_ = 45 °C and *RH*_THC_ = 35%).

The temperature variation profile for the moisture desorption process is shown in Fig. 14. The desorption of moisture from the desiccant solution to the air resulted in a decrease in the temperature of the samples compared to the temperature of the chamber caused by the latent heat of evaporation.

**Fig. 15:**
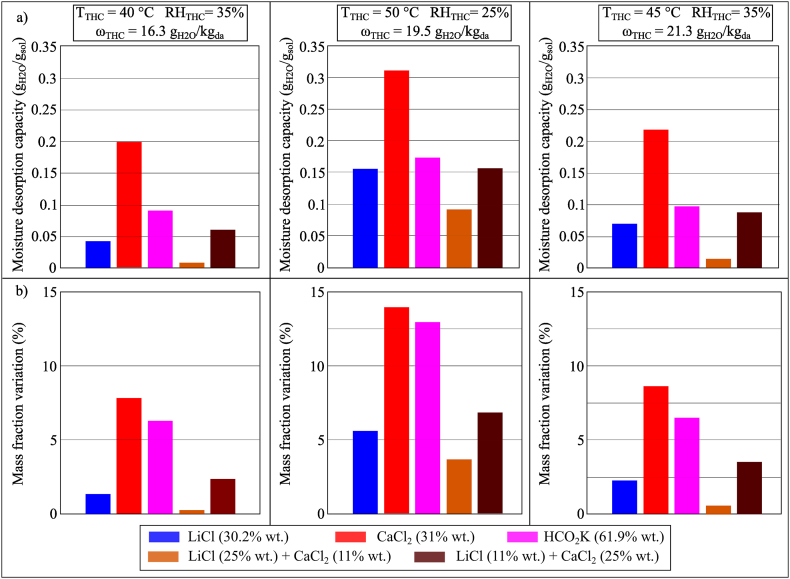
(a) MDC and (b) mass fraction variation of conventional desiccant solutions and mixtures during the moisture desorption process after 100 min in Conditions 1 (*T*_*THC*_ = 40 °C, *RH*_*THC*_ = 35%), 2 (*T*_*THC*_ = 50 °C, *RH*_*THC*_ = 25%) and 3 (*T*_*THC*_ = 45 °C, *RH*_*THC*_ = 35%).

The moisture desorption performance of LiCl, CaCl_2_, HCO_2_K, and mixtures of LiCl and CaCl_2_ (two different ratios) in three conditions of the temperature and humidity chamber were compared, as shown in Fig. 15(a and b). Compared to the moisture absorption process, the results showed that the moisture desorption process would be more affected by *T*_THC_ rather than *ω*_THC_. In Condition 2 (*T*_THC_ = 50 °C, *RH*_THC_ = 25%), aqueous CaCl_2_ solution offered the highest MDC, 0.3113 g_H2O_/g_sol_. HCO_2_K (61.9% wt.) presented a slightly higher MDC than that of a mixture of LiCl and CaCl_2_ (11% wt./25% wt.). However, similar to the findings reported in Section 4.1.1, with a concentration of water in the solution lower than 50%, HCO_2_K produced a larger mass fraction change between the beginning and the end of the moisture desorption process for the same amount of moisture desorbed, which would affect the capacity to store thermo-chemical energy of the desiccant solution as further discussed in Section 4.3.

#### 4.2.2 Addition of surfactant

**Fig. 16:**
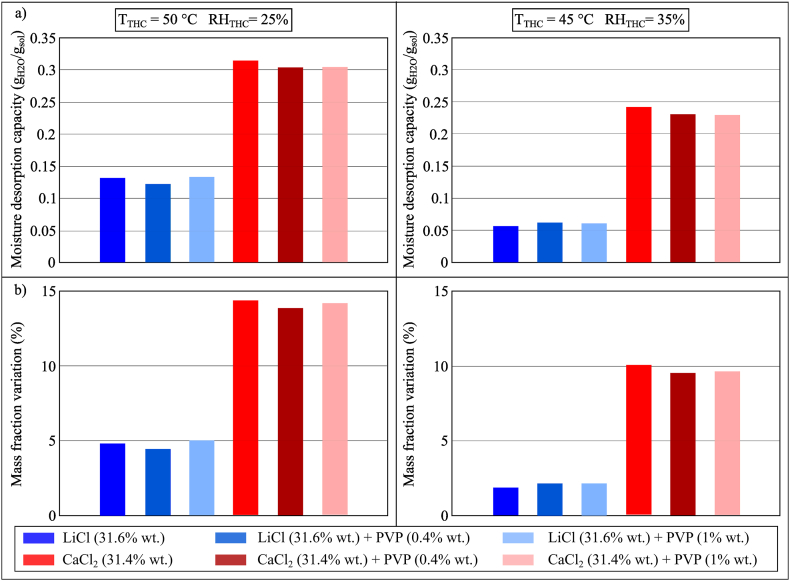
(a) MDC and (b) mass fraction variation of conventional desiccant solutions and PVP-K30 during the moisture desorption process after 100 min in Conditions 2 (*T*_*THC*_ = 50 °C, *RH*_*THC*_ = 25%) and 3 (*T*_*THC*_ = 45 °C, *RH*_*THC*_ = 35%).

The moisture desorption performance of LiCl and CaCl_2_ with and without PVP-K30 (0.4% and 1% wt.) were compared in Fig. 16(a and b). Similar to what was observed for the moisture absorption process, the addition of PVP-K30 would not have a significant effect on the performance of the desorption process. The type of base desiccant fluid would determine the moisture desorption capacity of the investigated sample, as evidenced by the higher moisture desorption capacity of CaCl_2_ samples compared to LiCl samples, presumably because the moisture desorption analysis is mainly governed by the equilibrium vapour pressure and equilibrium moisture content of the desiccant solutions.

#### 4.2.3 Addition of nanoparticles

**Fig. 17:**
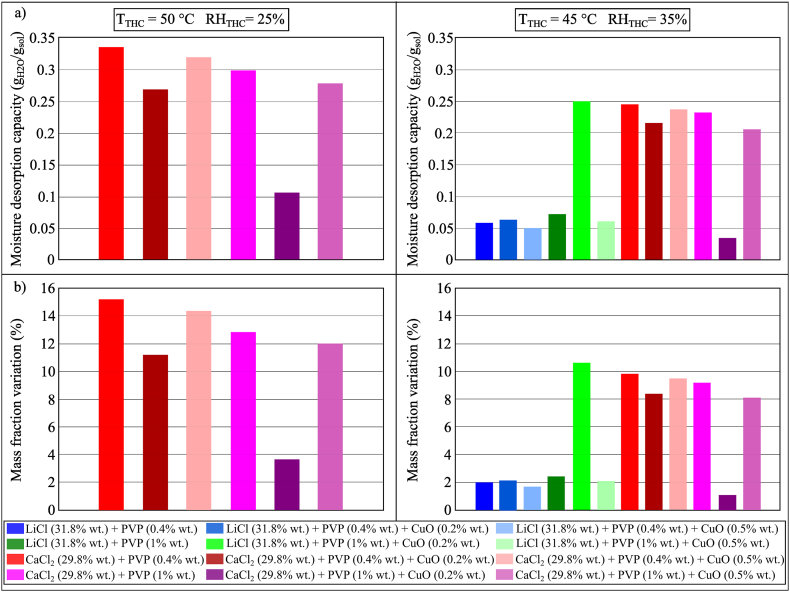
(a) MDC and (b) mass fraction variation of conventional desiccant solutions, PVP-K30 and CuO during the moisture desorption process after 100 min in Conditions 2 (*T*_THC_ = 50 °C, *RH*_THC_ = 25%) and 2 (*T*_THC_ = 45 °C, *RH*_THC_ = 35%).

The moisture desorption performance of LiCl and CaCl_2_ added with PVP-K30 (0.4% wt. and 1% wt.) with and without CuO (0.2% wt. and 0.5% wt.) were investigated, and the results are shown in Fig. 17(a and b). For most of the samples, a reduction in the moisture desorption performance was observed when CuO NPs were added. Compared to other CaCl_2_ samples, the mixture of CaCl_2_ (31.6% wt.), PVP-K30 (1% wt.) and CuO (0.2% wt.) (which offered a higher moisture absorption performance in Fig. 10) showed a significant drop in the moisture desorption performance. On the other hand, compared to other LiCl samples, the mixture of LiCl (29.8% wt.), PVP-K30 (1% wt.) and CuO (0.2% wt.) (which presented a lower moisture absorption performance in Fig. 10) showed a significant increase in the moisture desorption performance and became comparable to that of the CaCl_2_ samples. This implied that the addition of NPs could enhance the moisture desorption process but the stability of the produced nanofluid might determine its moisture desorption capacity.

#### 4.2.4 Ionic liquids

**Fig. 18:**
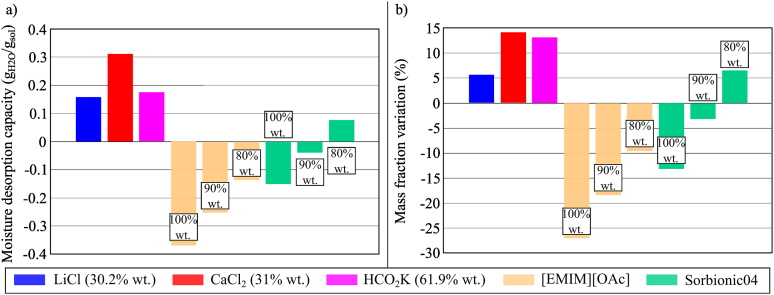
(a) MDC and (b) mass fraction variation of conventional desiccant fluids, [EMIM][OAc], and Sorbionic04 during the moisture desorption process after 100 min in Condition 2 (*T*_THC_ = 50 °C, *RH*_THC_ = 25%).

**Fig. 19:**
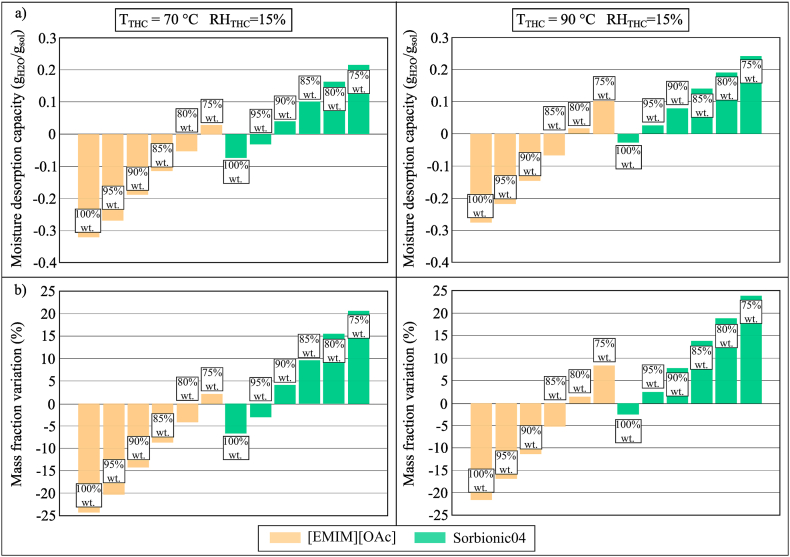
(a) MDC and (b) mass fraction variation of [EMIM][OAc] and Sorbionic04 during the moisture desorption process after 100 min in Conditions 4 (*T*_THC_ = 70 °C, *RH*_THC_ = 15%) and 5 (*T*_THC_ = 90 °C, *RH*_THC_ = 15%).

The moisture desorption performance of [EMIM][OAc] (80% wt., 90% wt., and 100% wt.) and Sorbionic04 (80% wt., 90% wt., and 100% wt.) were compared to that of LiCl (30.2% wt.), CaCl_2_ (31% wt.) and HCO_2_K (61.9% wt.). The results in Fig. 18(a and b) indicated that when the temperature and humidity chamber was set to 50 °C and 25% RH, except for Sorbionic04 (80% wt.), all [EMIM][OAc] and Sorbionic04 samples would not desorb moisture but absorbed moisture from the air. Based on experimental results, only Sorbionic04 (80% wt.) showed the potential to be regenerated when its temperature ranged between 40 and 44.4 °C. To further evaluate the desorption capacity of [EMIM][OAc] and Sorbionic04 at higher temperatures, the moisture desorption tests were repeated at 70 °C and 90 °C while keeping the RH at 15%. The results are shown in Fig. 19(a and b), showing different behaviour during the moisture desorption of [EMIM][OAc] and Sorbionic04. For [EMIM][OAc], higher temperatures were required for regeneration. When the temperature and humidity chamber was 70 °C and 15% RH, only [EMIM][OAc] (75% wt.) was able to desorb moisture to the air but the performance was trivial (0.0276 g_H2O_/g_sol_) and the temperature of the sample could be as high as 58 °C. This indicated the potential of [EMIM][OAc] to perform moisture absorption (*i.e.* dehumidification) at relatively high temperatures, which could be beneficial for applications, such as industrial dryers [62] or automotive flash-off drying [63]. When the chamber was set to 90 °C and 15% RH, the moisture desorption performance of [EMIM][OAc] solution (75% wt.) increased to 0.1 g_H2O_/g_sol_ and the temperature of the [EMIM][OAc] solution reached 71.1 °C. On the other hand, the moisture desorption performance of Sorbionic04 would be better, offering MDC of 0.2154 g_H2O_/g_sol_ and 0.2421 g_H2O_/g_sol_ in Conditions 4 and 5 of the temperature and humidity chamber (70 °C, 15% RH and 90 °C, 15% RH, respectively), whereas the temperature of the sample reached 57.4 °C and 66.3 °C. In both Conditions 4 and 5, the temperature of Sorbionic04 samples was lower than that of [EMIM][OAc] samples.

**Fig. 20:**
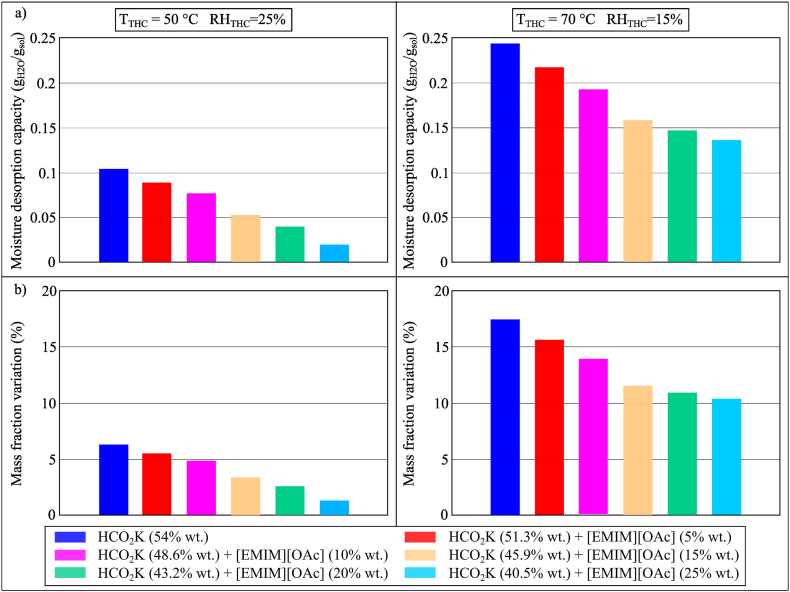
(a) MDC and (b) mass fraction variation of the mixtures of HCO_2_K and [EMIM][OAc] during the moisture desorption process after 100 min in Conditions 2 (*T*_THC_ = 50 °C, *RH*_THC_ = 25%) and 4 (*T*_THC_ = 70 °C, *RH*_THC_ = 15%).

The MDC and the mass fraction variation of the mixtures of HCO_2_K and [EMIM][OAc] in Conditions 2 (50 °C, 25% RH) and 4 (70 °C, 15% RH) were compared with that of aqueous HCO_2_K solution, as shown in Fig. 20(a and b). Unlike the findings shown in Figs. 12(a), Fig. 20(a) showed that the addition of [EMIM][OAc] to an aqueous HCO_2_K solution would reduce the MDC. When the concentration of [EMIM][OAc] increased from 0% wt. to 25% wt., the MDC of the solution in Conditions 2 and 4 of the temperature and humidity chamber would reduce from 0.104 g_H2O_/g_sol_ to 0.0198 g_H2O_/g_sol_ and from 0.244 g_H2O_/g_sol_ to 0.136 g_H2O_/g_sol_, respectively. This implied the need for a higher temperature to effectively desorb the moisture from the desiccant solution when [EMIM][OAc] was added to the aqueous HCO_2_K solution.

### 4.3 Economic and thermo-chemical energy storage capacity analysis

**Fig. 21:**
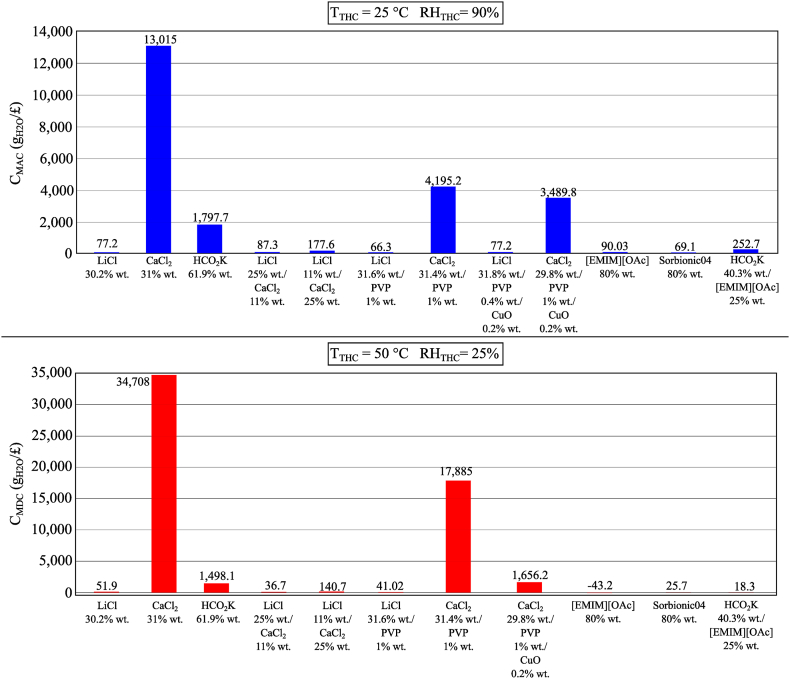
Trade-off between the moisture sorption process performance and the cost of the desiccant solution.

The trade-off between the performance of the moisture sorption process and the cost of the desiccant solution was evaluated using *C*_MAC_ and *C*_MDC_ defined in Section 3. The higher these values, the better the performance from a cost perspective. Fig. 21 presents the results of the analysis for the moisture absorption and desorption processes in two conditions of the temperature and humidity chamber (*T*_THC_ = 25 °C, *RH*_THC_ = 90%; and *T*_THC_ = 50 °C, *RH*_THC_ = 25%, respectively) for some selected samples of each of the analyzed categories of conventional and innovative desiccant solutions.

The low cost of CaCl_2_ strongly affects the economic performance of both the moisture absorption and desorption processes, ensuring a higher performance from a techno-economic point of view. However, it is worth mentioning that CaCl_2_ would not be able to absorb moisture from the air if *ω*_air_ is low, for example, when the temperature and humidity chamber is 25 °C and 70% RH as shown in Fig. 7. On the contrary, the high cost of LiCl limits its feasibility for moisture sorption from an economic point of view. Compared to LiCl, HCO_2_K is preferable as its cost is significantly lower whilst its moisture absorption capacity is similar to that of LiCl, which results in higher *C*_MAC_ and *C*_MDC_ (1797.7 g_H2O_/£ and 1498.3 g_H2O_/£ for HCO_2_K vs. 77.2 g_H2O_/£ and 51.9 g_H2O_/£ for LiCl, respectively). Mixtures of LiCl and CaCl_2_ show the potential to increase economic performance. For instance, *C*_MAC_ and *C*_MDC_ of LiCl/CaCl_2_ (11% wt./25% wt.) mixture are more than double that of LiCl. However, the lower moisture absorption capacity of the mixture compared to the stand-alone LiCl desiccant should be considered. When the mass fraction of LiCl increases, as in the mixture of LiCl (25% wt.) and CaCl_2_ (11% wt.), the economic benefits of using mixtures are reduced.

The high potential of using [EMIM][OAc] in liquid desiccant systems, in particular for deep dehumidification processes, is justified by its high moisture absorption capacity and higher *C*_MAC_ compared to LiCl (90.03 g_H2O_/£ vs. 77.2 g_H2O_/£). However, the feasibility of using this IL for low-grade heat recovery is affected by the higher temperature required for the moisture desorption process. On the other hand, Sorbionic04 shows similar performance as LiCl for the moisture absorption process and lower performance for the moisture desorption process. This implies that Sorbionic04 could be used as a replacement for LiCl in liquid desiccant systems with the advantages of not producing crystals and being non-corrosive at the expense of requiring a heat source of a slightly higher temperature for efficient regeneration of the desiccant solution. The addition of PVP-K30 to the desiccant salt would not have a significant effect on the moisture sorption process performance, while the cost of the surfactant would have a limited impact on the economic performance of the moisture sorption process. The high cost of CuO NPs (123.96 £/kg) would reduce the economic performance despite its small concentration.

**Fig. 22:**
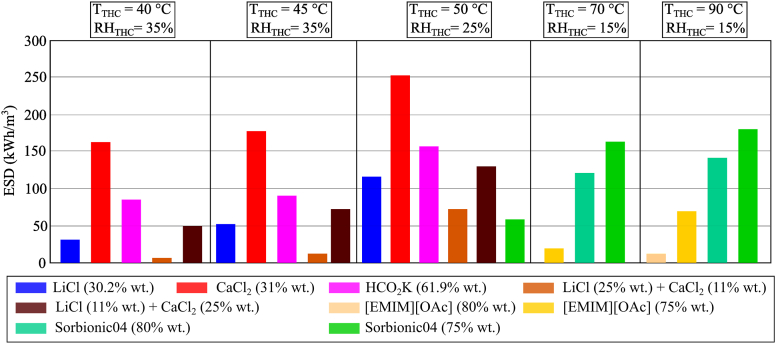
Volumetric energy density of desiccant fluids based on moisture desorption analysis.

When heat is supplied to a desiccant solution for regeneration (*i.e.,* moisture desorption), it increases the concentration of the desiccant solution, which can then be used for dehumidification (*i.e*., moisture absorption) without a further supply of thermal energy. In this regard, the concentrated desiccant solution stores thermal energy in the form of thermo-chemical energy/absorption potential. As such, a larger concentration glide is required for storing a significant amount of thermo-chemical energy in the liquid desiccant solution. The effect of temperature and humidity on the volumetric energy storage density, *ESD*, was investigated for some selected desiccant samples based on the results of the moisture desorption analysis. As shown in Fig. 22, the *ESD* of the CaCl_2_ solution could reach 252.6 kWh/m^3^ when the temperature and humidity chamber was 50 °C and 25% RH. This value indicated the high potential of CaCl_2_ for storing energy in the form of thermo-chemical energy compared to water (*ESD* of 69.4 kWh/m^3^ for a Δ*T* of 60 °C stored as sensible heat), underground materials, such as rock, dry and wet soil (*ESD* of 30 kWh/m^3^ for a Δ*T* of 50 °C stored as sensible heat), organic materials (*ESD* of 27.8–69.4 kWh/m^3^ stored as latent heat) and inorganic materials (*ESD* of 41.7–119.4 kWh/m^3^ stored as latent heat) [64]. In real operating liquid desiccant systems, low-flow technology is required to achieve a large concentration variation between the concentrated and the diluted solution [65]. Conventional technology usually involves high flow rates, which are required to limit the increase/decrease in the temperature of the desiccant solution while absorbing/desorbing moisture from/to the air. On the other hand, these internally cooled/heated systems use a lower solution flow rate, which allows achieving a larger difference in the solution concentration and, as such, storing higher thermo-chemical energy.

The results shown in Fig. 22 were estimated for the charging process of the thermo-chemical energy storage (*i.e.* moisture desorption). However, desiccant solutions capable of achieving a larger variation in concentration for the discharging process of the thermo-chemical energy storage would also be favoured for application in liquid desiccant systems since a lower concentrated solution (*i.e.*, higher *ω*_eq_) would have a higher performance during regeneration (*i.e.*, moisture desorption), as shown by HCO_2_K in Section 4.1.1. Similar to HCO_2_K, [EMIM][OAc] and Sorbionic04 could also work if the concentration of water in the solution was lower than 50%. This would allow achieving a larger mass fraction variation between the beginning and the end of the moisture absorption process for the same amount of moisture absorbed, resulting in a diluted solution with higher moisture desorption capacity.

## 5 Conclusion

This study presented an experimental and feasibility investigation of desiccant solutions (either conventional or innovative) based on moisture sorption and techno-economic analyses to enhance the current understanding of moisture absorption and desorption behaviour and assess the feasibility of using innovative fluids. CaCl_2_ showed low moisture absorption performance but high moisture desorption capacity (up to 0.3113 g_H2O_/g_sol_ in the climatic chamber at 50 °C and 25% RH, implying higher potential for low-grade heat recovery) and thermo-chemical energy storage capacity for charging (252.6 kWh/m^3^ under the same climatic chamber condition). Ionic liquids were proved as feasible replacements for conventional desiccant solutions with larger moisture absorption capacity, such as LiCl and HCO_2_K, due to their ability to provide high moisture absorption at relatively high temperatures, although these solutions showed a lower capacity to recover low-grade heat sources. A maximum moisture absorption capacity of 0.429 g_H2O_/g_sol_ was achieved for pure [EMIM][OAc] in the climatic chamber at 25 °C and 90% RH. Ionic liquids could be used as additives to increase the performance of conventional desiccant solutions without having a significant effect on cost. The addition of PVP-K30 and CuO nanoparticles showed limited or no effect on the performance of the moisture absorption or desorption process. The high cost of CuO nanoparticles could limit the feasibility of nanofluids in liquid desiccant systems. The current research offers insights into what desiccant solutions could have the best performance and lowest cost under various conditions. Both kinetics and sorption rates have not been investigated in this study, neither has the use of all innovative solutions been trialled in real operating liquid desiccant systems, which present limitations of this study. As ionic liquids appeared more promising as shown by the results gained in this study, future research should investigate kinetics, sorption rates, and testing of the use of ionic liquids, both stand-alone and as additives, in real operating liquid desiccant systems.

## Author contribution statement

Alessandro Giampieri: Conceived and designed the experiments; Performed the experiments; Analyzed and interpreted the data; Contributed reagents, materials, analysis tools or data; Wrote the paper.

Yngrid Machado: Performed the experiments; Analyzed and interpreted the data.

Janie Ling-Chin: Analyzed and interpreted the data; Contributed reagents, materials, analysis tools or data; Wrote the paper.

Anthony Paul Roskilly: Contributed reagents, materials, analysis tools or data; Wrote the paper.

Zhiwei Ma: Conceived and designed the experiments; Wrote the paper.

## Data availability statement

Data will be made available on request.

## Declaration of competing interest

The authors declare that they have no known competing financial interests or personal relationships that could have appeared to influence the work reported in this paper.
